# Study protocol: psychological and physiological consequences of exposure to mass media in young women - an experimental cross-sectional and longitudinal study and the role of moderators

**DOI:** 10.1186/s40359-014-0037-0

**Published:** 2014-09-20

**Authors:** Simone Munsch

**Affiliations:** Department of Psychology, Clinical Psychology and Psychtherapy, University of Fribourg, Fribourg, Switzerland

**Keywords:** Body image, Cognitive distortion, Emotion regulation, Eating behavior

## Abstract

**Background:**

Repeated exposure to thin beauty ideals is part of the daily routine. Exposure to thin ideals via mass media plays an important role in the development and maintenance of eating disorders (EDs), low self-esteem, depressive or anxious feelings in young females. It is important to elucidate the circumstances under which exposure to thin ideals develops its detrimental impact and to investigate whether these features are more pronounced in EDs than in other mental disorders also related to negative body image.

**Methods/design:**

We investigate the following key questions: (1) Does laboratory induced exposure to thin ideals (waiting room design) relate to impairments in terms of body image, affect and eating behavior and biological stress response (salivary alpha-amylase, salivary cortisol, heart rate and heart rate variability) in 18 to 35 year old female suffering from anorexia and bulimia nervosa (AN, BN) compared to female healthy controls and to a sample of females suffering from mixed mental disorders (depression, anxiety and somatic symptom disorder (SSD) disorders)? (2) How do moderators such as cognitive distortions (“Thought-Shape Fusion, TSF”), and correlates of emotion regulation (ER) moderate the influence of the exposure? (3) Are these characteristics amenable to change after treatment? Altogether 250 female participants including patients with AN, BN, depressive, anxiety and SSD disorders, and healthy women will be recruited in Switzerland and Germany.

**Discussion:**

The findings will provide knowledge about the role of moderators influencing the effects of exposure to thin ideals promoted by mass media in eating disorder (ED) patients, patients suffering from mixed mental disorders and healthy controls. Evaluating their differential susceptibility will contribute to a better understanding of the role of negative body image in the maintenance of not only symptoms of ED, but also of depression, anxiety and SSD. Additionally our results will shed light on the stability of effects in healthy controls as well as in the patient groups before and after treatment as usual. Findings foster the development of tailored interventions including a training in specific ER strategies as well as cognitive restructuring of distorted beliefs about the own body when confronted with thin ideals.

**Trial registration:**

German Clinical Trials Register: DRKS00005709. Date of registration: 6^th^ of February, 2014.

## Background

Body image is a multidimensional construct including subjective bodily and physical attitudes and experiences. Body image attitudes refer to an evaluative component such as self-ideal discrepancies and an investment component such as the salience of one’s appearance (Cash et al. 2004). According to Cash (Cash et al. 2004), certain situations activate schema-based processing of self-evaluative body image thoughts and affect-laden information about one’s appearance. A dysfunctional attitude towards one’s body during adolescence in females has been so pronounced that over the last 25 years it has been considered to be a normative discontent (Ricciardelli & McCabe 2004). Clinically significant levels of a negative body image represent constant stressors and are associated with low self-esteem, depressive, anxious and somatic symptom disorder (SSD) symptoms (Nishina et al. 2006; Rodgers et al. 2010; Martens et al. 2010). Dysfunctional attitudes towards one’s weight, shape and body size are further known to promote negative affect, restrictive dieting, self-induced vomiting, and abuse of laxatives, diuretics, diet pills and exercise (Stice et al. 2011).

Appearance and status among others comprise important aspects of everyday life. Mass media provides daily, multiple messages influencing social and individual norms regarding attractiveness, ideal body and shape, self-control, desire, food and weight management (Dittmar et al. 2006). It is a well-known fact that young females often try to attain unnatural and unhealthy body shapes, frequently transmitted by media (British Medical Association (BMA) 2000). In non-clinical students’ samples, detrimental consequences of exposure to the thin ideal included disturbed eating behavior, depressive feelings and low self-esteem. All have been shown to emerge after only 15 minutes of reading beauty magazines (Tiggemann 2003; Cameron & Ferraro 2004; Turner et al. 1997). This underlines findings revealing a larger impact of magazine viewing on body image dissatisfaction and disturbed eating behavior than television viewing. Nevertheless certain individuals seem even to feel better after having been exposed to a thin ideal (Mills et al. 2002).

Factors influencing the susceptibility towards the effect of thin ideal exposure promoted by mass media include comparison processes, the tendency to internalize thin body ideals promoted by media (Tiggemann 2003), (Lockwood & Kunda 1997; Myers et al. 1992; Mussweiler et al. 2000; Myers & Crowther 2009) and cognitive distortions such as consistent, non-veridical and skewed thinking often found in different mental disorders (Shafran & Robinson 2004). In *ED research*, the concept of “Thought-Shape Fusion, TSF” (Radomsky et al. 2002), was developed according to the “thought-action fusion” concept in individuals with obsessional compulsive disorders (Shafran et al. 1996; Shafran et al. 1999). The concept includes *likelihood TSF*, referring to the irrational belief, that simply thinking about eating a forbidden food makes it likely that a person can gain weight or change shape. *Moral TSF* describes the belief that thinking about a forbidden food is as morally wrong as eating the food. *Feeling TSF* refers to the phenomenon that experiencing thoughts about eating forbidden food increases the feeling of fatness: “I feel fatter after thinking about eating forbidden foods (e.g. chocolate)” (Shafran & Robinson 2004). TSF can be induced in individuals suffering from EDs and controls resulting in stronger negative feelings and feelings of fatness, guilt and a perceived higher degree of moral wrong-doing (Radomsky et al. 2002; Shafran et al. 1999; Coelho et al. 2008; Coelho et al. 2010). It has not yet been investigated, whether analogously to the thinking about food, merely thinking about ideal bodies may induce cognitive distortions such as TSF.

Another factor probably influencing the susceptibility towards thin ideals promoted by mass media is the capacity to regulate emotions. The concept of emotion regulation (ER) encompasses strategies to regulate emotional experience and relies on the capability to correctly perceive, recognize, identify and express emotions (Thompson 1994; Gross 2007; Haynos & Fruzzetti 2011). A broad data base indicates that individuals who are not able to engage in effective management of emotional responses to everyday events experience longer and more severe periods of distress that may evolve into diagnosable depression or anxiety (Aldao et al. 2010). There is increasing data underlining the important role of ER in EDs (Tice et al. 2001; Guerrieri et al. 2008). Especially AN and to a somewhat lesser extent BN individuals seem to be affected by difficulties in identifying and labeling basic emotions (Harrison et al. 2010) compared to healthy controls. These problems are associated with more frequent use of maladaptive coping strategies (Harrison et al. 2010). According to the results of a current meta-analytic review (Haynos & Fruzzetti 2011) deficient ER correlates are found even after treatment of EDs and these deficiencies might be related to relapses. Nevertheless, it is an open question, whether these ER particularities are a correlate of acute ED episodes (the starving state) or whether they persist during remission (Oldershaw et al. 2012).

**Figure 1:**
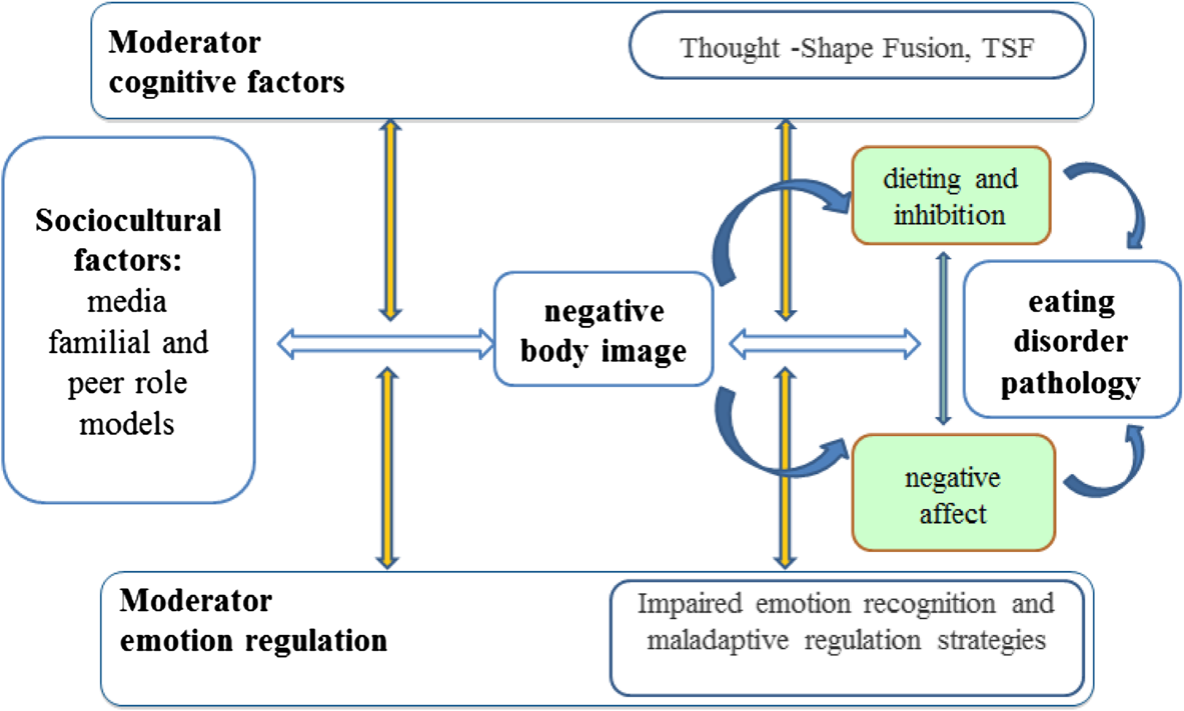
**Maintenance of eating disorder.**

Figure 1 summarizes maintenance factors of disordered eating behavior according to Stice and colleagues (Stice et al. 2011) and highlights the moderators, which will be investigated in the current study. Other factors influencing the susceptibility towards the effects of media exposure will be considered as covariates (p. 16).

We assume that daily exposure to unachievable thin ideals and the resulting negative effects represent a moderate daily stressor and fulfills the criteria of egoinvolvement (Matias et al. 2011; Kirschbaum & Hellhammer 1989; Jacobs et al. 2007). The effect might be comparable to the experience of repeated moderate stress. Stress response in terms of cortisol release has been shown to be altered even though preserved on a higher level after standardized stressors in AN. BN patients cortisol response to stressors is similarly to healthy controls (Lo Sauro et al. 2008; Zonnevylle-Bender et al. 2005; Kirschbaum et al. 1993; Monteleone et al. 2011). The Hypothalamic-pituitary-adrenal axis (HPA) in BN individuals was normally activated, however salivary alpha-amylase concentrations were increased.

In this context, the goals of the present study are as follows: To investigate the differential susceptibility to the effects of an exposure to magazines promoting the thin ideal (thin ideal) versus neutral magazines (neutral) on body image, affect, eating behavior and biological stress response in groups of female patients suffering from AN and BN or mixed mental disorders and a healthy control group. Additionally the stability of these effects will be examined in all groups. Another main focus lies on the role of the moderators ER capacity and cognitive distortion type (TSF) regarding their effect on differential susceptibility to exposure to thin ideals.

## Methods/design

### Hypotheses

- 1.1 Exposure to *thin ideal* in contrast to *neutral* magazines causes an impairment of body image (FRS, VAS_B), affect (threeAS), eating behavior (VAS_E) and a physiological stress response (increase in mean salivary cortisol and alpha-amylase concentrations, HR and decrease in HRV).
- 1.2 Exposure to *thin ideal* in contrast to *neutral* magazines is related to more pronounced negative body image (FRS, VAS_B), negative affect (threeAS), disordered eating behavior (VAS_E) and physiological stress response (increase in mean salivary cortisol and alpha-amylase concentrations, HR and decrease in HRV) in individuals with AN, BN, and the clinical control group (depression and SSD disorders) compared to the healthy control group, both at T1 and less at T2.
- 1.3 Exposure to *thin ideal* in contrast to *neutral* magazines is related to more pronounced negative body image (FRS, visual VAS_B), negative affect (threeAS), disordered eating behavior (VAS_E) and physiological stress response (increase in mean salivary cortisol and alpha-amylase concentrations, HR and decrease in HRV) in individuals with AN and BN compared to the clinical control group, both at T1 and less at T2.
- 1.4 The impact of thin ideal exposure on negative body image (FRS, VAS_B), negative affect (threeAS), disordered eating behavior (VAS_E) and physiological stress response (increase in mean salivary cortisol and alpha-amylase concentrations, HR and decrease in HRV) is moderated by cognitive style (TSFstate_B and/or TSFtrait_B) and ER capacity (see measures for detailed description). We expect stronger responses for participants with high TSF and low ER capacity.

### Study design

This study is a multi-site cross- and longitudinal experimental trial examining the impact of exposure to the thin ideal (body image, affect, eating behavior, psychophysiological stress-response) using a standardized laboratory waiting room design (Turner et al. 1997). The design favors daily real life situations (high ecological validity) and foregoes a computerized presentation of thin ideals. Participants suffering from AN and BN vs. patients with mixed mental disorders vs. healthy control groups (between subject factor) are tested during a media exposure and during a non-media exposure condition (between and within subject factor) over time (Pre-Post; within subject factor) in order to examine stability of expected effects three months later. This time span corresponds to an approximated mean treatment duration for AN and BN patients in collaborating Swiss and German clinics. Treatment components are assessed based on the German evidence based guidelines for diagnosis and treatment (Herpertz et al. 2011). Treatment components are double checked by therapists and patient-ratings (Bandelow et al. 2013; DGPPN et al. 2009; Schaefert et al. 2012; Becker et al. 2013).

### Participants

**Figure 2:**
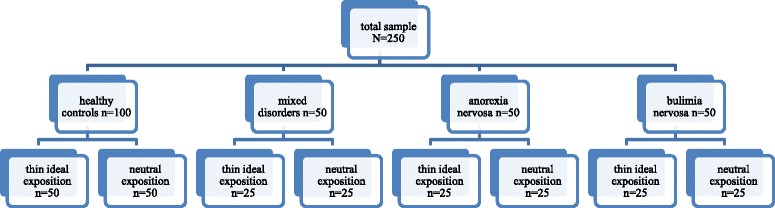
**Study sample.**

Altogether 250 participants will be included in the study. Healthy participants (N = 100) as well as participants suffering from AN (N = 50), BN (N = 50) or mixed mental disorders (depressive, SSD and anxiety disorders; N = 50) will be randomly distributed to either a thin ideal or neutral exposition based on magazine viewing (Figure 2). All participants will be evaluated regarding the presence of any mental disorder during face to face interview based on the Structured Clinical Interview for mental disorders currently adapted for DSM-5 by the Silvia Schneider group, DIPS for DSM-5 (see Table 1 for an overview of all interviews and psychological questionnaires).

**Table 1:** **Instruments**

**Instrument Or**.: **original version**, **Ge**.: **German translation**	**Description**/**Construct**	**Part of the study**
**Interviews**		
**Diagnostic interview for psychiatric disorders**, (DIPS) (Schneider & Margraf 2011)	Structured interview to assess psychiatric disorders according DSM-IV-TR, according to DSM-5 in prep. by S. Schneider et al.	Diagnostic phase
**Structured Clinical Interview for DSM**-**IV** Axis I, Section G, Body Dysmorphic Disorder (SKID I) (Wittchen et al. 1997)	Structured interview to assess psychiatric disorders according DSM-IV	Diagnostic phase
**Self**-**Report Inventory**		
**Psychopathology**/ **Mood**		
**Beck Depression Inventory II** (BDI-II), Or. (Beck et al. 1996); Ge. (Hautzinger et al. 2009)	21 items; measures severity of depressive symptoms	Baseline
**Beck Anxiety Inventory** (BAI), Or. (Beck et al. 1988); Ge. (Margraf & Ehlers 2007b)	21 items; measures severity of anxiety	Baseline
**Three Dimensions Affect Scale** (ThreeAS) (Wihelm & Schoebi 2007)	6 items; measures the basic mood-dimensions valence, calmness, and energetic arousal scale	During the experiment
**Screening for Somatoform Disorders** (SOMS-7 T) (Rief & Hiller 2008)	53 items; covers all somatic symptoms mentioned as occurring in somatization disorder, according to DSM-IV and ICD-10	Baseline
**Body Dysmorphic Dysorder Questionnaire** (BDDQ), Or. (Phillips 1998) Ge. (Bohne et al. 2002)	4 items, measures the preoccupation with an imagined or slight defect in appearance, which is not better accounted for by another mental disorder	Baseline
**Self**-**Esteem**		
**Rosenberg Self**-**Esteem**-**Scale** (RSES), Or. (Rosenberg 1965); Ge. (Collani & Herzberg 2003)	10 items; measures a general state self-esteem	Baseline
**Eating Behavior**		
**Dutch Eating Behavior Questionnaire** (DEBQ), Or. (Van Strien et al. 1986); Ge. (Grunert 1989)	10 items (subscale emotional eating), measures eating in response to emotional states	Baseline
**Eating Disorder Examination Questionnaire** (EDE-Q), Or. (Fairburn & Beglin 1994); Ge. (Hilbert & Tuschen-Caffier 2006)	28 items; 4 scales: eating concerns, weight concerns, restraint eating, shape concerns; assessment of relevant characteristics of eating disorders that have occurred during the past 28 days	Baseline
**VAS eating** (VAS_E), in prep. by Munsch et al.	7 items; assessment of eating behavior (desire to binge, to purge and to restrict)	During the experiment
**Body Image**		
**Figure Rating Scale** (FRS) (Stunkard et al. 1983)	9 figures of increasing body size (very thin to very obese), to assess body image satisfaction by calculating diff. between current image and ideal image	During the experiment
**Sociocultural Attitudes Towards Appearance Questionnaire** (SATAQ), Or. (Heinberg et al. 1995); Ge. (Knauss et al. 2009)	16 items; 3 subscales: internalization of the media body ideal, perceived pressure from the media and awareness of the body ideal	Baseline
**Thought**-**shape Fusion Trait Scale** – **short version** (Trait TSF Short), Or. (Coelho et al. 2013); Ge. in prep. by Munsch et al.	18 items; 2 subsections: 14 items trait TSF, 4 items clinically relevant food-related thoughts; 3 components likelihood, feeling and moral	Baseline
**Body Image Thought**-**shape Fusion Trait Scale** (TSFtrait_B), in prep. by Munsch et al.	30 items; 3 components likelihood, feeling and moral, assessment of thin ideal related cognitions	Baseline
**Body Image Thought**-**shape Fusion State Scale** (TSFstate_B), Or. (Radomsky et al. 2002); Ge. in prep.by Munsch et al.	10 items; questionnaire to assess aspects of thought-shape fusion, e.g. feelings of anxiety and guilt	During the experiment
**VAS body image** (VAS_B), in prep. by Munsch et al.	11 items; assessment of satisfaction with the appearance and body-related emotions	During the experiment
**Appereance Schemas Inventory**-**Revised** (ASI-R), Or. (Cash & Labarge 1996); Ge. (Grocholewski et al. 2011)	20-item, including two factors: Self-Evaluative Salience and Motivational Salience	Baseline
**Body Image Satsifacion Scale** (BIS), Or. (Turner et al. 1997); Ge. in prep. by Munsch et al.	Short version with 12 items: body image satisfaction, dieting attitudes/behaviors, preoccupation with thinness	Baseline
**Emotion regulation**		
**Difficulties in Emotion Regulation Scale** (DERS), Or. (Gratz & Roemer 2004); Ge. (Ehring et al. 2010)	36 items; 6 dimensions intended to characterize central aspects of affective experience and emotion processing	Baseline
**Barratt impulsiveness Scale** – short version (BIS-15), Or. (Patton et al. 1995); Ge. (Meule et al. 2011)	15 items, assessment of impulsivity, 3 subscales (non-planning impulsivity, motoric impulsivity, attention-based impulsivity)	Baseline
**Emotion regulation State Scale** (VAS_Emo), in preparation by Munsch et al.	7 items, measures different emotional regulation strategies during the experiment	During the experiment
**Social Comparison Processes**		
**Physical Appearance Comparison Scale** (PACS), Or. (Thompson et al. 1991); Ge. in prep. by Munsch et al.	Individual’s tendency to compare their own appearance to the appearance of others in social situations	Baseline & during Experiment
**Third**-**Person Perception**		
**Third Person Perception** (TPP), Or. (David et al. 2009); Ge. . in prep. by Munsch et al.	4 items; 2 scales: 2 Items perceived effect of pictures on self, 2 items perceived effect of picture on other women (third person)	During the experiment
**Post Event Processing**		
**Post Event Processing** (PEPQ), Or. (Rachman et al. 2000); Ge. in prep. by Munsch et al.	13 items, measures the effects of pictures in a media exposure during an experiment 24 h later	After the experiment

Measures will be taken before, during and after the waiting room paradigm. This will be followed by a period of three months during which the participants in the clinical groups (AN, BN, clinical control) will be treated as usual whereas the healthy control group remains untreated. At the end of the treatment period remission status will be assessed (remission: “not meeting all criteria for an AN or BN at the time of discharge, BMI >18.5 and a global EDE-Q score of less than 2.3; partial remission: “weight gain of more than half of the target weight gain in order to achieve a BMI of 18.5, reduction of 30% of the initial eating disorder pathology (EDE-Q), 30% reduction of binge eating and compensatory episodes; no remission: “Less than 50% of the target weight gain, less than 30% reduction of the initial eating disorder pathology (EDE-Q), less than 30% reduction of binge eating and compensatory episodes” (Zipfel et al. 2014; Stice et al. 2013; Agras et al. 2000). Remission status for the mixed mental disorders group will be approximated as follows: Depression: “not meeting all criteria for a depressive disorder at the time of discharge, BDI-II score ≤ 12 (Riedel et al. 2010); partial remission: reduction of 30% of the initial BDI-II score; no remission: less than 30% reduction of the initial BDI-II score. Anxiety: “not meeting all criteria for any anxiety disorder at the time of discharge; BAI score of ≤ 15 (Margraf & Ehlers 2007a; Goldschmidt 2008), partial remission: reduction of 30% of the initial BAI score, no remission: less than 30% reduction of the initial BAI score. SSD: “not meeting all criteria for a SSD at the time of discharge, SOMS complaints score less than 3 (Rief & Martin 2014), partial remission: reduction of 30% of the initial intensity SOMS score; no remission: less than 30% reduction of the initial SOMS score (Rief & Martin 2014). Additionally therapists will assess improvement during the treatment course using the Clinical Global Impressions Scale, CGI (Busner & Targum 2007).

The type of treatment, the specific treatment interventions, the intensity of treatment as well as supplementary interventions such as body-oriented trainings are assessed according a self-developed assessment scale. This scale is based on evidenced-based treatment guidelines according to Herpertz and colleagues (Herpertz et al. 2011) and was adapted in order to allow parallel assessment in treatment settings in both Swiss and German collaborating clinics.

### Inclusion criteria

- Age 18 to 35 years of age

### Informed consent

- Diagnoses of AN or BN based on DSM-5 criteria (eating disorder group)
- Diagnoses of either depressive, SSD or anxiety disorders (mixed mental disorders group) based on DSM-5 criteria *and* absence of a current ED, EDE-Q general score below 2.5
- Healthy control group: absence of present mental disorder *and* EDE-Q general score below 2.5 ^a^ (Fischer et al. 2012).

### Exclusion criteria

- Pregnancy or lactation
- Psychotic or bipolar and related disorders
- Serious medical conditions having an effect on eating and mood.
- Participation in another trial
- Lack of compliance with study procedure
- Current intake of weight-affecting drugs
- Past bariatric surgery

### Ethical approval

The study was approved by the ethical committee of the leading center at the University of Fribourg (no. 2012_001) and by the ethical committee of the canton of Fribourg (023/12-CER-FR) as well as in the cantons of collaborating clinics in Switzerland. In Germany, the study was approved by the ethical committee at the University of Bochum. Written informed assent and consent in accordance with the Declaration of Helsinki will be obtained (Declaration of Helsinki). All procedures within this research project will be conducted in accordance with the guidelines for Good Clinical Practice (GCP) by clinically trained investigators under the permanent supervision of the main applicants (International Conference on Harmonisation). All data will be coded without personal identifiers to ensure confidentiality. Participants may withdraw from the trial at any point without any penalty. A compensation of CHF 250,-/ 200 EURs will be offered for full participation in the project.

### Recruitment

Recruitment of age matched female patients takes place in clinical units in Switzerland and Germany. At all clinical units, all incoming patients from target groups (AN, BN, mixed mental disorder group) will routinely be asked for their agreement to participate. Recruitment of healthy controls takes place at the University of Fribourg, Switzerland and at includes students of Psychology as well as students of colleges of professional schools in the canton of Fribourg.

### Procedure

**Figure 3:**
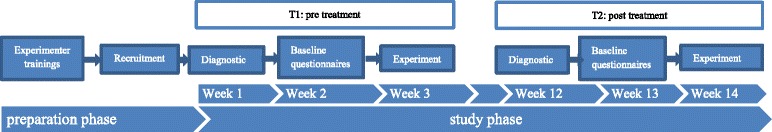
**Times schedule.**

During week 1 diagnostic interviews take place and participants will be randomized to either the thin ideal exposure or the control condition of the waiting room paradigm. In week 2 all participants receive a link for internet-based administering of questionnaires (www.umfrageonline.com). The waiting room paradigm takes place in week 3 respectively week 14 between 2 and 4.20 p.m. Study language is German. Refer to Table 1 for all instruments and Figure 3 for the time schedule of the trial.

### Experimental procedure

- 1. Preparation phase: During the preparation phase of 35 minutes, participants are asked to dispense the first saliva sample, to run the emotion recognition task (*decode*_*EMO*), to put on the ECG belt to assess HR, to fill in the sociodemographic questionnaire (Munsch et al. 2007) and self-report measures of body image, affect and eating behavior (*pre media exposure*).

Assessment of biological measures: Measurement of salivary cortisol and alpha-amylase concentrations during and after media exposure will be performed 9 times. The sampling protocol was chosen to capture peak and recovery of salivary alpha-amylase, as well as salivary cortisol (Kirschbaum & Hellhammer 1989; Nater & Rohleder 2009). The participants will be instructed to place the cotton roll in a specific area of the mouth for 2 minutes and chew on it and to avoid physical exercise, eating or drinking anything but water, brushing teeth or smoking for at least 1 hour before examination. Medication, somatic and mental diseases, date of last menstruation, last alcohol consumption and habitual smoking will be documented. HR will be measured continuously using ambulatory monitoring systems such as Movisens (www.movisens.com). Belts will be put on at the beginning of the experimental procedure and will be worn during the whole procedure. To minimize artifacts (e.g. orthostatic reaction) we analyze HR as well as HRV after a rest of two minutes after changing the position (e.g. going from room 2 into room 3). Start and end points of different stages of the experiment will be marked using corresponding software.

**Figure 4:**
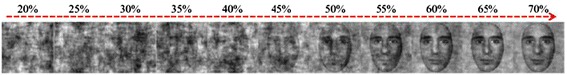
**Decode_EMO.**

Assessment of emotion recognition: The emotion recognition task, which is part of the multilevel assessment of ER capacity, is based on a computerized assessment determining the *quantity* of information that is necessary to each individual observer to achieve effective decoding of a facial expression of emotion (*decode*_*EMO*) (Miellet et al. 2011). The example (Figure 4) illustrates how phase-coherence of the very same individual (image) depicting a Westerner posing with a neutral expression is manipulated. The approach is coupled with QUEST (Watson & Pelli 1983), a psychometric method that allows the rapid estimation of a psychophysical threshold. Altogether 20 male and 20 female faces from the Karolinska Directed Emotional Faces (KDEF) face database (Lundqvist et al. 1998), displaying the 6 facial expressions (i.e., happy, sad, fear, anger, surprise, disgust) plus neutral are used.

**Figure 5:**
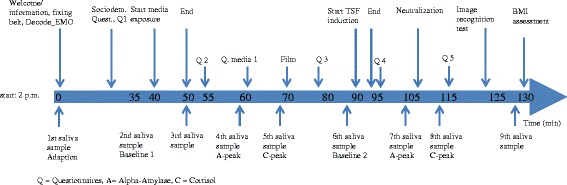
**Experimental procedure.**

- 2. Waiting room design: Afterwards the 2nd saliva sample is collected (baseline cortisol, amylase; *pre media exposure*). The participants are asked to leave personal belongings in the first room and will be guided into the standardized waiting room. In accordance to an adapted design of the waiting room study of Turner et al. (Turner et al. 1997) the participants will be asked to wait while baseline heart rate measures are assessed. The experimenter leaves the room and returns 3 minutes later with one magazine. Participants are explicitly told to have a close look at the pictures in the magazine while waiting. The experimenter puts the magazine on the table in front of the participant and leaves the room for 10 minutes. The magazine will be either a beauty magazine promoting the thin ideal (fashion magazine) or one carefully chosen neutral magazine (nature magazine). Spring issues of the magazines will be presented in study centers and will be replaced once a year. No other reading materials or pictures of people will be available in the waiting room. After 10 minutes participants are asked to move to the first room again. This room consists of a table, some chairs and a laptop (to fill in the questionnaires). The 3rd saliva sample is then collected (*post media exposure*) and participants are asked to complete the same self-report questionnaires (*post media exposure*). Then the 4th saliva sample is collected (*post media exposure*). Thereafter there is a recovery time between media exposure and TSF induction. During this time span participants fill in a questionnaire regarding their mass media use and the 5^th^ saliva sample is collected. Additionally an 8 minute nature film is presented in order to bridge the time between the end of the media exposure and TSF induction. In 14 patients and 45 healthy participants, the film did not impact on mood (p > .3), with the exception of a slight reduction of bad mood (Questionnaire media Zimmermann & Wirth, in preparation).
- 3. TSF Induction: After sampling of the 6th saliva sample and completing questionnaires (*pre TSF Induction*), TSF is induced (Radomsky et al. 2002; Shafran et al. 1999; Coelho et al. 2008): Participants in the thin ideal exposure group will be asked to imagine the female bodies in the magazine which they considered to be most attractive (*TSF induction*) in vivid detail, including height, weight, breast, hip, legs, arms, etc. for a 5 minutes period. If they did not read the magazine they are instructed to imaging attractive bodies in general as described above. In the neutral condition they are asked to imagine landscape pictures in the magazine that they liked most. Participants are then asked to write down the sentence: “I am imagining…” (describing the female body or the landscape respectively they are imaging (Radomsky et al. 2002)). After completing the TSF induction participants are asked to fill in the same questionnaires plus the body_TSFstate and the 7th salvia sample is collected (*post TSF induction*/ *pre neutralization*). At this point participants have the opportunity to *neutralize* their feelings during 5 minutes (Shafran & Robinson 2004; Shafran et al. 1999). Examples of neutralization activities will be provided including exercising (e.g. jumping jack), body checking, drawing pictures or mental neutralization (e.g. imagining to exercise, counting, etc.). The experimenter takes note whether the participant chose to neutralize the statements. The 8th salvia sample is collected and the questionnaires plus body_TSFstate completed (*post neutralisation*) (Figure 5 illustrates the experimental procedure).

Manipulation check: The implicit picture recognition test at the end is a custom script written in MatLab, using the Psychophysics Toolbox extensions (Brainard 1997; Pelli 1997). Subjects have to judge whether they have previously seen the picture by use of two keys on a standard keyboard. In both conditions, subjects are presented with ten images from the magazine used in the media exposure and ten randomly selected images from similar magazines. Pictures are presented until a response is given to ensure full recognition and encoding (reaction time; see (Grill-Spector & Kanwisher 2005)) and hit rate (%-correct recognition) as well as d’ (a measure for sensitivity) are used to calculate subject’s performance. At the end of the experiment BMI is measured, the HR belt is removed and the 9th salvia sample is collected. A short relaxation session is offered where necessary.

### Disclosure and debriefing

At pretreatment, participants are informed that they participate in a study investigating psychological well-being and psychophysiological responses to daily stressors in young women. After the experiment, they are asked not to disclose the purpose of the study to potential participants. They will be debriefed 3 months later, after the second performance of the experiment and receive a summary of the overall study aim together with preliminary findings as well as an overview of the results of their personal data entries.

### Measures

#### Primary and secondary outcomes

Primary outcome is the impact of thin ideal exposure on body image, affect and eating behavior (for an overview and psychometric properties of interviews and questionnaires, refer to Table 1). The effect of exposure and subsequent TSF induction will be determined by self-rating, visual analogue scales questionnaires (VAS_B, FRS, threeAS, VAS_E).

Secondary outcomes include the psychophysiological stress response induced by thin ideal exposure which is assessed by salivary concentrations of cortisol, alpha-amylase and heart rate (HR) and heart rate variability (HRV).

### Moderators

The ER index includes the capability to decode emotional facial expressions (decode_EMO) self-reported correlates of impulsivity (BIS-15), ER strategies (DERS) measured prior to the experiment and of physiological markers such as (HR and HRV) measured during the whole laboratory waiting room design. Cognitive factors are assessed by the trait variable “thought shape fusion” TSFtrait_B, and the intensity of TSFstate_B.

### Covariates

Depending on the specific hypothesis, analyses will be adjusted for covariates: baseline values of depression (BDI-II) and anxiety (BAI); eating disorder pathology (EDE-Q, DEBQ); socio-economic status, (SES); BMI, internalization of thin ideal (SATAQ-G); trait and correlates of acute appearance schema activation (ASI-R); SSD symptoms (SOMS) self-esteem (RSES), presence of comorbid mental disorders (DIPS). We will further take into account the role of Social Comparison Processes (PACS) and Third-Person Perception (TPP), which has been shown to influence the effects of media exposure in healthy young individuals in media psychology research.

### Methodological aspects

#### Power analysis

Sample size calculations are based on findings of small to medium effects of mass media engagement on body image in young adults from the general population (Levine & Murnen 2009) and based on findings from our pilot study, using the software G*Power, version 3.13 (Faul et al. 2007), and assuming two-sided tests with Alpha = .05 and Beta = 0.2 (Power = 0.8). Hypotheses H-1.1 and H-1.2 assuming changes in the subjective and physiological state due to the exposure to the thin ideal correspond to the main effect experimental condition on pre-post exp.-differences. In the pilot study these changes were moderate to large for the subjective states (affect, body image). Assuming such an effect size in the population (*Cohen*’*s f* > 0.30, which can be computed from ɳ^*2*^) the required sample size would be 90, (45 in each condition) to ensure a power > .80. Changes in the physiological variables were small in the pilot study (*f* < .16) and would require a larger sample size of at least 309 participants. However, the experimental manipulation is further qualified. H-1.2 predicts that effects of the thin ideal induction are stronger for patients than for non-patients. This is indicated by an interaction effect (group * condition; contrast: healthy vs. patients), which was small in the pilot study (*f* < .18). Thus the total sample size should be at least 245. H-1.3 predicts differences between the clinical groups and indicated by an interaction effect too (group * condition; contrast: BN + AN vs. clinical control). Due to the rather small number of patients in the pilot study, different diagnostic groups were not compared to each other. Differences between patients with eating disorders and the clinical comparison group are assumed to be small to moderate and thus sample requirements will be similar. With a sample of size of N = 250 also small to medium moderator effects (H-1.4) (Figure 1) could be detected.

To obtain the required number of 150 patients, taking into account a participation rate of 50% and a dropout rate of 30% in the patient population, a total of 390 patients (c. 130 in each patient group) have to enter the clinical units during the two years of assessment. Thus the recruitment of 195 patients will cover two consecutive years and will be continuous to prevent seasonal effects. In order to achieve the sample size suggested by the power analysis, a sample of 100 healthy controls in Fribourg (50 university students and 50 students of professional schools) is recruited.

### Randomization

All participants who meet criteria and who give informed consent are randomized to the experimental condition (exposure to fashion magazines) or control condition (exposure to neutral magazines) based on the randomized block/split-plot design approach (Lane 2008). Randomization is performed at the study center at the Department of Psychology in Fribourg. The randomization is stratified by age. The allocation ratio between the two conditions is 1:1.

### Blinding

Interviews are performed by two doctoral students in Switzerland and Germany under the supervision of the respective post doc and the principal and co-investigator. Interrater reliability is routinely assessed and guaranteed (kappa no lower than .60). Blinding of different, independent interviewers is not feasible, as this would impede clinical routine of the collaborating clinics. Interviewers and experimenters have no academic or therapeutic relationships with the participants. Computerized versions of self-reports and psychological questionnaires are filled in by patients individually during the experiments.

### Data analytic plan

The design of the study refers to a mixed four-way factorial ANOVA with exposure type (“exposure”, two levels) and study group (“group”, four levels) as between-subjects, and pre-post-exposure phase (“prepost-exposure”, two levels) and pre-post-intervention phase (“prepost-treatment”, two levels) as within-subjects factors. This model may be simplified to a three-way ANOVA by analyzing the two time points pre- and post-treatment separately. It can further be simplified to a two factorial model if, the pre-post-exposure difference is analyzed, using the pre values as a covariate (Vickers & Altman 2001). Additional further covariates can be added to the model if necessary. In the case of hypothesis 1.4, the principle model contains the factors exposure and prepost-exposure plus the moderator of concern, while the factor group can be added if required. Note that we will use linear mixed models to analyze the data as these types of models have been shown to have more statistical power to detect actually existing study effects and to lead to less biased results, in the case of dropouts, relative to models based on the randomized block/split-plot design approach (Lane 2008).

### Monitoring and data management

Data will continuously be monitored for completeness, consistency and plausibility by each of the study centers in Bochum, Germany and Fribourg, Switzerland under the lead of the main study center in Fribourg. Data entry will be double-checked. Data quality is ensured based examination of ranges. Data on longitudinal effects will only be released after study completion (after experiment 2). Besides the cross-sectional data no preliminary analysis of the longitudinal data is planned. Study data will be reported in accordance to the Consort guidelines (Moher et al. 2001).

### Safety aspects

Adverse events are not expected. Nevertheless any aggravating of symptoms even when not related to the experimental procedure will be documented at every assessment throughout the study procedure.

## Discussion

By elucidating the role of moderators influencing the effect of the exposure to the thin ideal in the maintenance of negative body image, affect and disordered eating and stress response, the results will provide evidence of the effect of thin ideal exposure in different groups of health and participants suffering from EDs and other mental disorders. Understanding the role of cognitive distortions and ER particularities in EDs and other mental disorders will help to specify interventions aiming at the restructuring of irrational beliefs about eating, weight and shape and to further develop the training of specific components of ER. Developing treatment modules encompassing the ability to express and tolerate emotions, as well as the ability to correctly identify and recognize emotions in significant others might be linked to increased remission rates in AN and in BN patients, where interpersonal functioning is known to maintain the disorder (Oldershaw et al. 2012; Arcelus et al. 2013). Additional knowledge on physiological consequences of exposure to thin ideals will help to understand stress reactivity in EDs and other mental disorders when confronted with moderate ego-involvement daily stressors.

Comparing EDs with other clinical conditions known to be related to a negative body image over time will further allow to specify whether body image disturbances are a general feature of psychopathology or whether they are most pronounced in EDs and whether this susceptibility remains stable or whether it is amenable to current treatment as usual. Finally, enhanced understanding of processes involved in the effect of exposure to thin ideals promoted by mass media in young females will guide understanding of possible vulnerability factors.

### Trial Status

This study is ongoing and will continue until January 2017.

### Endnote

^a^Based on our data obtained from 1500 young Swiss adolescents from a university and a general population we expect the value of general eating disorder pathology measured by EDE-Q general score above 2 to be associated with increased depressiveness and social stress.

## Abbreviations

AN: Anorexia Nervosa
ANOVA: Analysis of Variance
ASI-R: Appearance Schemas Inventory-Revised
BAI: Beck Anxiety Inventory
BDI-II: Beck Depression Inventory II
BIS-15: Barratt impulsiveness Scale
BMI: Body Mass Index
BN: Bulimia Nervosa
CGI: Clinical Global Impressions Scale
CHF: Swiss Francs
DEBQ: Dutch Eating Behavior Questionnaire
Decode_EMO: Decoding emotional expressions
DERS: Difficulties in Emotion Regulation Scale
DIPS: Diagnostisches Interview für psychische Störungen/ Diagnostic interview for psychiatric disorders
DSM-5: Diagnostic and Statistical Manual of Mental Disorders – Fifth Edition
ED: Eating Disorder
EDE-Q: Eating Disorders Examination Questionnaire
ER: Emotion Regulation
EUR: Euro
FRS: Figure Rating Scale
GCP: Good Clinical Practice
HPA: Hypothalamic-pituitary-adrenal
HR: Heart Rate
HRV: Heart Rate Variability
KDEF: Karolinska Directed Emotional Faces
PACS: Post Event Processing
RSES: Rosenberg Self-Esteem-Scale
SATAQ-G: Sociocultural Attitudes Towards Appearance Questionnaire German
SES: Socio-economic status
SOMS: Screening for Somatoform Disorders
SSD: Somatic symptom disorder
T1: Time point 1
T2: Time point 2
ThreeAS: Three Dimension Affect Scale
TPP: Third Person Perception
TSF: Thought-Shape Fusion
TSFstate_B: Thought-Shape Fusion State Scale Body
TSFtrait_B: Thought-Shape Fusion Trait Scale Body
VAS_B: Visual Analog Scale Body Image
VAS_E: Visual Analog Scale Eating
